# Glycemic variability and muscle loss in elderly type 2 diabetes: insights from continuous glucose monitoring and chest CT in 303 patients

**DOI:** 10.3389/fendo.2026.1863226

**Published:** 2026-06-22

**Authors:** Xiao-le Zhang, Ning Bai, Chun-pu Mao, Chun-yan Liu

**Affiliations:** Department of Endocrinology, Affiliated Hospital of Jiangnan University, Nanjing, China

**Keywords:** continuous glucose monitoring, elderly, glucose variability, sarcopenia, type 2 diabetes mellitus

## Abstract

**Purpose:**

Sarcopenia is common among older adults with type 2 diabetes mellitus (T2DM). Although chronic hyperglycemia is known to contribute to muscle loss, it remains unclear whether glucose fluctuations—independent of average glucose levels—also play a role. This study aimed to investigate the association between glucose fluctuation and pectoralis muscle mass in elderly patients with T2DM.

**Methods:**

This retrospective cross-sectional study included 303 elderly patients with T2DM who underwent continuous glucose monitoring and chest CT between October 2022 and December 2025. Glycemic variability was assessed using several metrics: coefficient of variation (CV), standard deviation (SD), mean amplitude of glycemic excursions (MAGE), mean of daily differences, time in range, time above range, time below range (TBR), and mean blood glucose. Pectoralis muscle index (PMI) was measured from CT images at the T4 vertebra level. Multivariable linear regression, subgroup analyses, and sensitivity analyses were performed.

**Results:**

Four glycemic variability indicators—CV, SD, MAGE, and TBR—were independently associated with lower PMI after adjusting for confounders (all *P* < 0.05). Among these, CV explained the largest proportion of PMI variance (adjusted R² = 0.22), followed by TBR (0.21), SD (0.20), and MAGE (0.19). A linear dose-response relationship was observed between CV and PMI (*P* for trend < 0.001). The inverse association was stronger in patients with diabetes duration ≥ 10 years (β = –0.18, *P* < 0.001) and those with BMI < 24 kg/m² (β = –0.18, *P* < 0.001), with significant interactions for both subgroups (*P* = 0.022 and 0.027, respectively). Sensitivity analyses confirmed the robustness of these findings.

**Conclusions:**

Greater glycemic variability is independently associated with lower pectoralis muscle mass in elderly patients with T2DM, especially among those with longer disease duration or normal body weight. Stabilizing glucose fluctuations may be an important consideration for preserving muscle mass in this population.

## Introduction

Population aging has led to a growing overlap between type 2 diabetes mellitus (T2DM) and older age (1), with the global prevalence of T2DM among older adults rising at an alarming rate. When the two conditions come together, patients face substantially higher risks of frailty, falls, functional decline, and death—not to mention a marked drop in quality of life (2). Therefore, it is increasingly important to identify modifiable factors that contribute to muscle loss in older adults with T2DM.

Traditional markers like hemoglobin A1c (HbA1c) provide an average picture of glucose levels over time, but they do not capture short-term glucose fluctuations (3). Over the past decade, researchers have started paying more attention to glucose variability (GV)—that is, both day-to-day and within-day fluctuations—as an independent risk factor for diabetic complications, separate from mean glucose levels, as evidenced by recent large-scale studies (4–6). It is well established that T2DM affects muscle through multiple pathways, including insulin resistance, chronic inflammation, and mitochondrial dysfunction (7). Notably, experimental evidence suggests that oscillating glucose may be more damaging to skeletal muscle than sustained hyperglycemia, possibly because it triggers greater oxidative stress and disrupts the signaling pathways needed for muscle protein synthesis (8). With continuous glucose monitoring (CGM) now being used more widely in clinical practice, it is possible to quantify GV using a range of metrics, thereby providing a more detailed view of glycemic control than HbA1c alone (9–11).

When it comes to measuring muscle mass, dual-energy X-ray absorptiometry and bioelectrical impedance analysis are still the standard tools (12). But in real-world clinical settings, many older patients end up getting chest CT scans for other reasons—say, to check for pulmonary infections or for cancer follow-up. That creates an opportunity to do opportunistic body composition analysis without extra radiation or cost. The pectoralis muscle index (PMI), measured from a single axial CT slice at the T4 level, has been shown to correlate well with total skeletal muscle mass and to predict outcomes in various diseases (13, 14). This approach allows for retrospective assessment of muscle mass without additional radiation exposure or cost, making it practical for large-scale studies.

Despite advances in both glycemic assessment and body composition measurement, the relationship between CGM-derived glycemic variability and CT-based pectoralis muscle mass in older adults with T2DM remains poorly understood. Very few studies have looked at whether GV is independently tied to muscle loss in this population after accounting for average glucose levels and other known risk factors. We hypothesized that greater glycemic variability would be independently associated with lower PMI, even after adjusting for mean glucose levels and other known risk factors.

To address this gap, we retrospectively analyzed 303 older patients with T2DM, integrating comprehensive CGM data with chest CT-derived PMI measurements. Our aim was to investigate the association between glucose fluctuation and pectoralis muscle mass, with the hypothesis that greater glycemic variability would be independently associated with lower PMI. The findings may provide new insights for risk stratification and prevention of diabetic sarcopenia in the aging population.

## Methods

### Study design and participants

We conducted a single-center, retrospective cross-sectional study. Participants were elderly patients with type 2 diabetes mellitus who were admitted to the Department of Endocrinology at the Affiliated Hospital of Jiangnan University between October 2022 and December 2025.

The inclusion criteria were: (1) age ≥ 60 years; (2) diagnosis of T2DM according to the 1999 World Health Organization (WHO) criteria (15) (fasting plasma glucose ≥ 7.0 mmol/L, 2-hour post-load glucose ≥ 11.1 mmol/L, or random glucose ≥ 11.1 mmol/L with classic symptoms); (3) underwent 72-hour CGM during hospitalization; (4) underwent CT scan within one week before or after CGM; (5) complete demographic and clinical data were available.

The exclusion criteria were: (1) other types of diabetes (e.g., type 1 diabetes, gestational diabetes, secondary diabetes); (2) severe hepatic or renal dysfunction (e.g., eGFR < 30 mL/min/1.73m², or liver enzymes > 3 times the upper limit of normal); (3) diagnosed malignancy, chronic wasting diseases (e.g., tuberculosis, hyperthyroidism), or long-term bedridden status; (4) use of medications affecting muscle metabolism (e.g., systemic glucocorticoids) within the past 3 months; (5) poor CT image quality that precluded accurate measurement of pectoralis muscle area.

After screening, 303 patients were included in the final analysis. Of the 303 included patients, the mean age was 69.77 years (range: 60–91 years), and 154 (50.83%) were male. Detailed baseline characteristics are shown in **Table 1**. The study protocol was approved by the hospital’s Ethics Committee (X202603) and followed the principles of the Declaration of Helsinki; informed consent was waived given the retrospective design.

**Table 1:** Baseline demographic, clinical, and key laboratory characteristics according to PMI tertiles.

Variables	Total (n = 303)	Lower tertile (n = 101)	Middle tertile (n = 101)	Upper tertile (n = 101)	P value
Demographic and clinical characteristics					
Age (years)	69.77 ± 7.14	69.90 ± 7.38	70.10 ± 6.20	69.31 ± 7.79	0.515
Male, n (%)	154.00 (50.83%)	33.00 (32.67%)	46.00 (45.54%)	75.00 (74.26%)	<0.001
BMI (kg/m²)	24.43 ± 3.04	24.73 ± 3.08	24.21 ± 2.94	24.35 ± 3.12	0.653
Diabetes duration (years)	12.85 ± 8.02	13.17 ± 8.14	12.55 ± 8.73	12.82 ± 7.19	0.872
Smoking history, n (%)	33.00 (10.89%)	10.00 (9.90%)	7.00 (6.93%)	16.00 (15.84%)	0.117
Comorbidities, n (%)					
Hypertension	204.00 (67.33%)	70.00 (69.31%)	69.00 (68.32%)	65.00 (64.36%)	0.730
Coronary heart disease	63.00 (20.79%)	26.00 (25.74%)	13.00 (12.87%)	24.00 (23.76%)	0.053
Diabetic retinopathy	73.00 (24.09%)	30.00 (29.70%)	16.00 (15.84%)	27.00 (26.73%)	0.053
Diabetic nephropathy	124.00 (40.92%)	49.00 (48.51%)	34.00 (33.66%)	41.00 (40.59%)	0.100
Medication history, n (%)					
Insulin use	202.00 (66.67%)	74.00 (73.27%)	61.00 (60.40%)	67.00 (66.34%)	0.152
Glycemic control					
FPG (mmol/L)	7.60 ± 2.94	7.46 ± 3.03	7.67 ± 2.91	7.68 ± 2.89	0.752
HbA1c (%)	8.82 ± 2.02	9.08 ± 2.01	8.76 ± 2.04	8.61 ± 2.00	0.122
Nutritional/Muscle-related markers					
Albumin (g/L)	40.29 ± 4.00	39.52 ± 4.73	40.69 ± 3.61	40.66 ± 3.46	0.070
Hemoglobin (g/L)	130.59 ± 16.03	128.85 ± 14.25	127.87 ± 16.14	135.06 ± 16.78	0.001
SCr (μmol/L)	74.75 ± 32.20	70.67 ± 28.64	76.05 ± 37.45	77.54 ± 29.71	0.024
Pectoralis muscle index					
PMI (cm²/m²)	18.72 ± 4.69	14.17 ± 1.59	18.19 ± 0.90	23.79 ± 3.98	<0.001

*Data are mean ± SD or n (%). P values from one-way ANOVA, Kruskal–Wallis, or chi-square test. Extended laboratory and full medication details are in **Supplementary Table 1**. BMI, body mass index; FPG, fasting plasma glucose; HbA1c, glycated hemoglobin; PMI, pectoralis muscle index.

### Sample size

This was a retrospective study, and the sample size was determined by the availability of eligible patients during the study period. As a *post-hoc* power consideration, following the common rule of thumb for multiple linear regression (10–20 events per variable), and with roughly 15 candidate variables in our final models, a sample size of 200–300 would be adequate. Our sample of 303 thus provided sufficient statistical power for the planned analyses.

### Data collection

We extracted demographic and clinical data from electronic medical records, including age, sex, body mass index (BMI), diabetes duration, smoking and alcohol use, medication history, and comorbidities (hypertension, coronary heart disease, diabetic retinopathy, diabetic nephropathy, diabetic peripheral neuropathy, and diabetic peripheral vasculopathy). Fasting laboratory measurements were collected after an overnight fast and included fasting plasma glucose (FPG), HbA1c, lipid profile (total cholesterol, triglycerides, LDL-C, HDL-C), hemoglobin, liver enzymes (AST, ALT), total bilirubin, serum uric acid, prealbumin, serum creatinine (SCr), blood urea nitrogen (BUN), cystatin C, retinol-binding protein, transferrin, serum iron, β2-microglobulin, neutrophil gelatinase-associated lipocalin (NGAL), thyroid-stimulating hormone (TSH), calcium, phosphorus, C-peptide, and albumin (ALB).

### Assessment of glucose fluctuation

All patients underwent at least 72 hours of continuous glucose monitoring using the FreeStyle Libre H system (Abbott), which records interstitial glucose levels every 5 minutes. CGM data were uploaded to and processed using the iZhangkong cloud-based diabetes management platform (Fuzhou Kangwei Network Technology Co., Ltd., Fuzhou, China; https://dm.izhangkong.com/).

The following glycemic control and fluctuation parameters were calculated: (1) mean blood glucose (MBG): the average of all glucose readings; (2) standard deviation (SD): the standard deviation of all glucose readings, reflecting overall dispersion; (3) Coefficient of Variation (CV): calculated as (SD/MBG) × 100%, representing glucose variability relative to the mean; (4) mean amplitude of glycemic excursions (MAGE): calculated using the method proposed by Service et al., averaging the arithmetic means of blood glucose increases or decreases that exceeded one standard deviation of the mean glucose value (16); (5) mean of daily differences (MODD): calculated as the absolute difference between glucose values at the same time on two consecutive days, averaged over the monitoring period. MODD specifically reflects inter-day glycemic variability; (6) time in range (TIR): Percentage of time spent with glucose levels within the target range (3.9–10.0 mmol/L); (7) time above range (TAR): Percentage of time spent with glucose levels above the target range (> 10.0 mmol/L); (8) time below range (TBR): Percentage of time spent with glucose levels below the target range (< 3.9 mmol/L) (11).

### Assessment of CT-derived pectoralis muscle index

Chest CT scans were acquired using a 64-detector row scanner under standardized protocols. Images were retrieved from the Picture Archiving and Communication System in DICOM format. Two radiologists, blinded to clinical data, independently measured pectoralis muscle area—a measure previously validated against whole-body skeletal muscle mass (17, 18).

For each patient, we selected a single axial slice at the fourth thoracic vertebral level. The pectoralis major and minor muscles were manually traced bilaterally, and the software computed the cross-sectional area (CSA, cm²). We used the average CSA of both sides and calculated the pectoralis muscle index (PMI) as the total CSA divided by height squared (m²).

Although PMI at T4 correlates with total skeletal muscle mass, it should be noted that a single axial slice may not fully capture whole-body muscle composition, and this limitation should be considered when interpreting the results.

### Bias control

Several measures were implemented to minimize bias. CT measurements were performed independently by two radiologists blinded to clinical data. Laboratory data came from standardized assays with routine quality control. CGM records were reviewed by two trained endocrinologists to ensure data integrity. Multivariable regression models adjusted for a broad range of potential confounders informed by prior literature and clinical reasoning. Subgroup and sensitivity analyses were used to assess the consistency and robustness of the findings.

### Statistical analysis

Statistical analyses were performed with SPSS (version 26.0, IBM Corp.). A two-tailed P < 0.05 was considered statistically significant. Continuous data are presented as mean ± SD for normally distributed variables or median (Q1, Q3) for non-normal distributions; categorical data as frequencies and percentages. Patients were divided into tertiles based on PMI: lower (< 16.62 cm²/m², n = 101), middle (16.62–19.80, n = 101), and upper (> 19.80, n = 101).

We used one-way ANOVA (or Kruskal–Wallis for non-normal data) to compare continuous variables across PMI tertiles, with Bonferroni correction for *post-hoc* comparisons, and chi-square tests for categorical variables. Normality was assessed using the Shapiro-Wilk or Kolmogorov-Smirnov test, guiding the choice of Pearson or Spearman correlation for univariate associations between PMI and clinical variables. For multivariable analysis, we built separate linear regression models for each glycemic variability parameter (CV, SD, MAGE, TBR) due to collinearity; each model adjusted for age, sex, BMI, diabetes duration, HbA1c, albumin, hemoglobin, and serum creatinine. Variables with P < 0.10 in univariate analysis or deemed clinically relevant were considered for entry.

Hierarchical regression was used to assess the incremental contribution of CV beyond traditional risk factors. To examine dose-response, we categorized CV into quartiles and computed adjusted mean PMI using ANCOVA. Subgroup analyses stratified by sex, age, BMI, diabetes duration, and HbA1c; interaction terms were added to regression models to test for effect modification. Sensitivity analyses included (1) excluding patients with extreme PMI values (< 1st or > 99th percentile), (2) excluding insulin users, and (3) additional adjustment for nutritional markers (prealbumin, transferrin). Intra- and inter-observer reproducibility for PMI measurements was evaluated using intraclass correlation coefficients (ICCs), interpreted per Koo and Li (19). Reporting followed the STROBE guidelines (20).

## Results

### Study population selection

We initially screened 1,960 patients with T2DM between October 2022 and December 2025. After excluding 820 patients under 60 years of age, 1,140 elderly patients (≥ 60 years) remained. Of these, 586 were excluded because they did not undergo CGM, and another 55 were missing chest CT examinations, leaving 499 patients who had both CGM and CT data available.

We then applied the exclusion criteria. A total of 196 patients were excluded for the following reasons: severe renal dysfunction (eGFR < 30 mL/min/1.73m², n = 28), severe hepatic dysfunction (liver enzymes > 3×ULN, n = 15), malignancy or chronic wasting diseases (n = 39), use of medications affecting muscle metabolism (n = 36), and poor CT image quality (n = 33). The final analysis included 303 patients.

**Figure 1:**
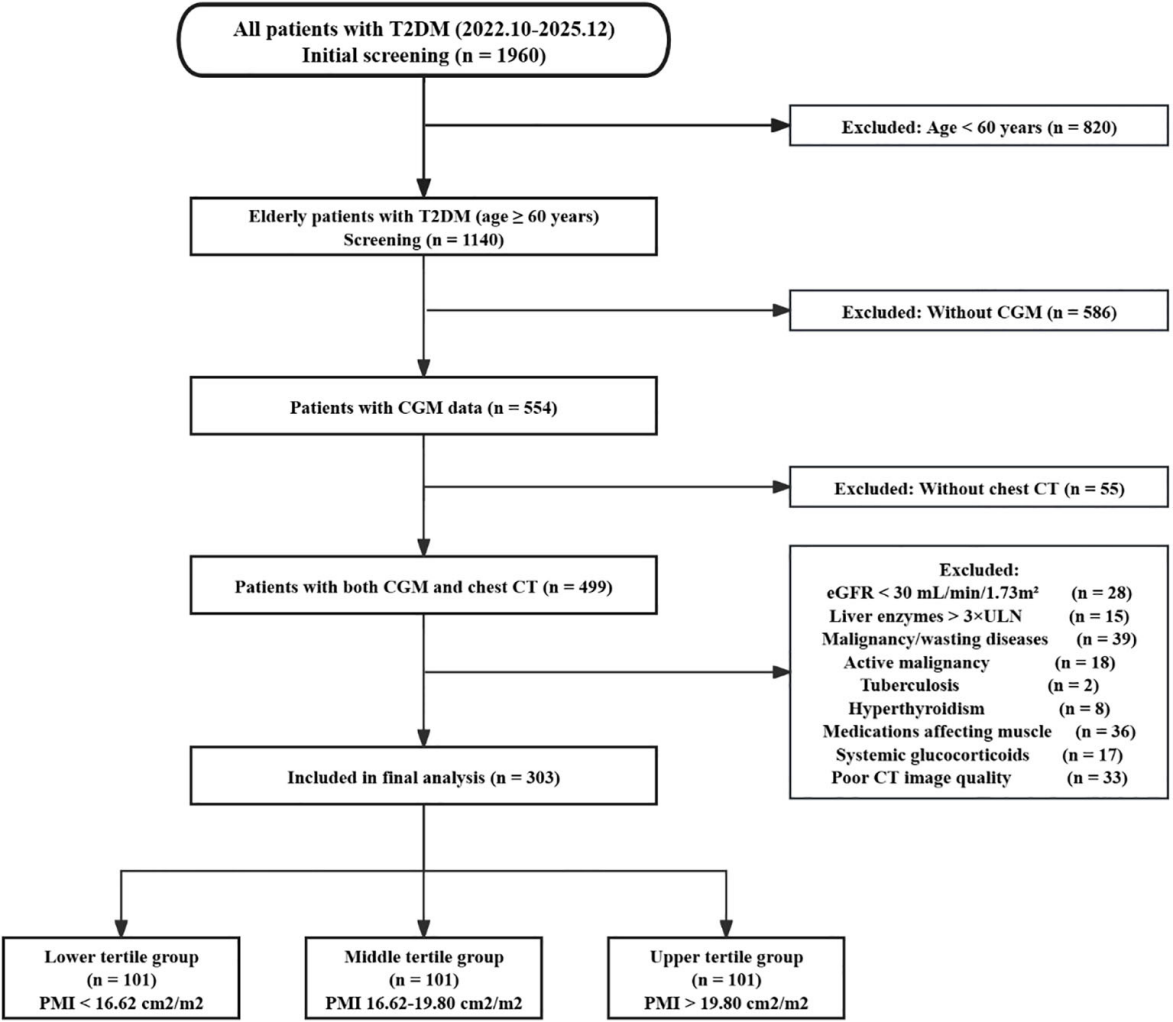
Flowchart of the study population selection process.

Based on PMI tertiles, we divided the patients into three groups: lower (PMI < 16.62 cm²/m², n = 101), middle (PMI 16.62–19.80 cm²/m², n = 101), and upper (PMI > 19.80 cm²/m², n = 101). **Figure 1** illustrates the selection process.

### Baseline characteristics and comparisons among PMI tertile groups

The baseline demographic, clinical, and laboratory characteristics of the 303 patients, as well as comparisons among the three PMI tertile groups, are presented in **Table 1** and **Supplementary Table 1**. The proportion of men rose progressively from the lower to the upper tertile (P < 0.001). Hemoglobin levels were highest in the upper tertile (P = 0.001), while serum creatinine was lowest in the lower tertile (P = 0.024). No significant differences were observed for age, BMI, diabetes duration, smoking or alcohol use, most comorbidities, medication use, or other laboratory measures (all P > 0.05).

### Characteristics of glucose fluctuation detected by CGM system

The comparisons of glucose fluctuation parameters among the three PMI tertile groups are shown in **Table 2**. Patients in the lowest PMI tertile had higher SD than those in the middle and upper tertiles (P = 0.002). CV followed a similar pattern: the lowest tertile had the highest values (33.50 ± 7.55%), and the highest tertile had the lowest (P < 0.001). MAGE was also higher in the lower tertile compared with the upper tertile (P = 0.023). TBR decreased progressively from the lowest to the highest PMI tertile (P = 0.031).

**Table 2:** Comparison of glucose fluctuation parameters among PMI tertile groups.

Variables	Total (n = 303)	Lower tertile (n = 101)	Middle tertile (n = 101)	Upper tertile (n = 101)	P value
MBG (mmol/L)	8.80 ± 1.99	8.71 ± 1.91	8.81 ± 2.18	8.89 ± 1.90	0.863
SD (mmol/L)	2.70 ± 0.90	2.97 ± 0.97	2.61 ± 0.88	2.53 ± 0.81	0.002
CV (%)	30.52 ± 7.27	33.50 ± 7.55	29.56 ± 6.77	28.49 ± 6.53	<0.001
MAGE (mmol/L)	5.20 ± 1.69	5.57 ± 1.76	5.09 ± 1.70	4.94 ± 1.55	0.023
MODD (mmol/L)	0.61 ± 0.49	0.68 ± 0.54	0.56 ± 0.46	0.59 ± 0.45	0.328
TIR (%)	66.21 ± 22.27	64.42 ± 18.68	67.63 ± 24.32	66.59 ± 23.49	0.198
TAR (%)	31.23 ± 22.94	31.71 ± 19.84	30.12 ± 24.93	31.87 ± 23.91	0.511
TBR (%)	2.54 ± 4.80	3.87 ± 6.48	2.22 ± 3.99	1.54 ± 2.94	0.031

*Data are presented as mean ± SD for normally distributed continuous variables and median (Q1, Q3) for non-normally distributed continuous variables. P values were calculated using one-way ANOVA for normally distributed variables and Kruskal–Wallis test for non-normally distributed variables. MBG, mean blood glucose; SD, standard deviation; CV, coefficient of variation; MAGE, mean amplitude of glycemic excursions; MODD, mean of daily differences; TIR, time in range; TAR, time above range; TBR, time below range.

### Correlation analysis between PMI and clinical variables

We assessed correlations between PMI and clinical variables using Pearson or Spearman correlation coefficients, depending on data distribution. As shown in **Table 3**, PMI correlated positively with male sex (r = 0.388, P < 0.001), hemoglobin (r = 0.18, P = 0.002), and serum creatinine (r = 0.17, P = 0.004). Among the glycemic variability metrics, PMI showed negative correlations with CV (r = –0.24, P < 0.001), SD (r = –0.20, P < 0.001), MAGE (r = –0.16, P = 0.005), and TBR (r = –0.13, P = 0.028).

**Table 3:** Correlation coefficients between PMI and selected clinical variables.

Variables	Correlation coefficient	P value
Age	-0.06	0.313
Male	0.388	<0.001
Diabetes duration	-0.02	0.751
Hemoglobin	0.18	0.002
Serum creatinine	0.17	0.004
MBG	-0.01	0.827
CV	-0.24	<0.001
MAGE	-0.16	0.005
MODD	-0.09	0.130
SD	-0.20	<0.001
TIR	0.09	0.107
TAR	-0.04	0.449
TBR	-0.13	0.028

Pearson or Spearman correlation coefficients were used as appropriate based on data distribution. MBG, mean blood glucose; SD, standard deviation; CV, coefficient of variation; MAGE, mean amplitude of glycemic excursions; MODD, mean of daily differences; TIR, time in range; TAR, time above range; TBR, time below range.

### Independent factors associated with PMI: multivariate linear regression analysis

We performed multivariable linear regression analyses to identify factors independently associated with PMI. Each model included age, sex, BMI, diabetes duration, HbA1c, albumin, hemoglobin, and serum creatinine, plus one glycemic variability parameter (CV, SD, MAGE, or TBR). We constructed separate models for each GV parameter to avoid issues with collinearity.

As shown in **Table 4**, all four GV parameters were independently associated with PMI after adjustment. In the respective models, CV (β = -0.14, 95% CI: -0.21 to -0.08, P < 0.001), SD (β = -1.02, 95% CI: -1.60 to -0.44, P < 0.001), MAGE (β = -0.39, 95% CI: -0.69 to -0.09, P = 0.01), and TBR (β = -0.18, 95% CI: -0.28 to -0.08, P < 0.001) each showed significant negative associations with PMI. Across all models, male sex was strongly and consistently associated with higher PMI (β range: 3.60–4.05, all P < 0.001). HbA1c was significantly associated with lower PMI in the CV and TBR models (P < 0.05 and P < 0.01, respectively), but not in the SD or MAGE models. Age, BMI, diabetes duration, albumin, hemoglobin, and serum creatinine were not independently associated with PMI in any model (all P > 0.05). The model including CV explained the greatest proportion of variance in PMI (adjusted R² = 0.22), followed by TBR (0.21), SD (0.20), and MAGE (0.19).

**Table 4:** Multivariable linear regression analyses for factors associated with PMI using different glycemic variability parameters.

Variable	Model with CV	Model with SD	Model with MAGE	Model with TBR
	β (95% CI)	β (95% CI)	β (95% CI)	β (95% CI)
Age (years)	-0.04 (-0.11, 0.03)	-0.04 (-0.11, 0.04)	-0.04 (-0.11, 0.03)	-0.04 (-0.11, 0.03)
Male sex	3.93 (2.82, 5.04)***	4.05 (2.92, 5.19)***	3.97 (2.83, 5.12)***	3.6 (2.48, 4.72)***
BMI (kg/m²)	-0.04 (-0.2, 0.12)	-0.01 (-0.08, 0.05)	-0.02 (-0.09, 0.04)	-0.03 (-0.09, 0.03)
Diabetes duration (years)	-0.02 (-0.08, 0.04)	-0.04 (-0.21, 0.12)	-0.04 (-0.2, 0.13)	-0.05 (-0.21, 0.12)
HbA1c (%)	-0.27 (-0.51, -0.03)*	-0.16 (-0.42, 0.10)	-0.24 (-0.49, 0.02)	-0.37 (-0.61, -0.12)***
Albumin (g/L)	0.09 (-0.05, 0.22)	0 (-0.04, 0.04)	0 (-0.04, 0.04)	0 (-0.04, 0.04)
Hemoglobin (g/L)	-0.01 (-0.04, 0.03)	0.1 (-0.04, 0.24)	0.11 (-0.03, 0.25)	0.11 (-0.03, 0.24)
Serum creatinine (μmol/L)	0 (-0.02, 0.01)	0 (-0.02, 0.01)	0 (-0.02, 0.01)	0 (-0.02, 0.02)
CV (%)	-0.14 (-0.21, -0.08)***	–	–	–
SD (mmol/L)	–	-1.02 (-1.6, -0.44)***	–	–
MAGE (mmol/L)	–	–	-0.39 (-0.69, -0.09)**	–
TBR (%)	–	–	–	-0.18 (-0.28, -0.08)***
Adjusted R²	0.22	0.20	0.19	0.21

*Data are presented as standardized β coefficients (95% confidence intervals). All models were adjusted for the listed covariates simultaneously. GV, glycemic variability; CV, coefficient of variation; SD, standard deviation; MAGE, mean amplitude of glycemic excursions; TBR, time below range. *P < 0.05, **P < 0.01, **P < 0.001, ***P < 0.001.

### Incremental value of glycemic variability in explaining PMI

We next performed hierarchical regression analyses to evaluate whether glycemic variability added explanatory value beyond traditional risk factors. We used CV as the primary GV indicator because it had the highest adjusted R² in the earlier models. As shown in **Table 5**, Model 1 included only demographic factors (age, sex, BMI) and accounted for 15% of the variance in PMI (adjusted R² = 0.15). Adding clinical and laboratory variables (diabetes duration, HbA1c, albumin, hemoglobin, serum creatinine) in Model 2 increased the adjusted R² to 0.17 (ΔR² = 0.02, P = 0.035). Including CV in Model 3 further improved the model fit, raising the adjusted R² to 0.22 (ΔR² = 0.05, P < 0.001)—meaning CV explained an additional 5% of the variance beyond the traditional risk factors.

**Table 5:** Hierarchical regression models showing incremental value of CV.

Model	Variables included	Adjusted R²	ΔR²	P for ΔR²
1	Age, sex, BMI	0.15	–	–
2	Model 1 + diabetes duration, HbA1c, albumin, hemoglobin, serum creatinine	0.17	0.02	0.035
3	Model 2 + CV	0.22	0.05	<0.001

### Dose-response relationship between glycemic variability and PMI

To examine whether there was a dose-response relationship, we divided patients into quartiles based on CV and calculated adjusted mean PMI using ANCOVA, adjusting for age, sex, BMI, diabetes duration, HbA1c, albumin, hemoglobin, and serum creatinine. As shown in **Table 6**, adjusted PMI decreased progressively across increasing CV quartiles (P for trend < 0.001), suggesting a linear inverse relationship.

**Table 6:** Dose-response relationship between glycemic variability and PMI (Adjusted PMI according to CV quartiles).

CV quartile	CV range (%)	n	Adjusted PMI (cm²/m²)*	P for trend
Q1	15.26 – 25.09	76	22.05 ± 0.42	<0.001
Q2	25.15 – 29.93	76	21.32 ± 0.41	
Q3	29.95 – 35.22	76	20.54 ± 0.41	
Q4	35.26 – 50.87	75	19.87 ± 0.42	

Values are estimated marginal means ± SE adjusted for age, sex, BMI, diabetes duration, HbA1c, albumin, hemoglobin, and serum creatinine.

### Subgroup analyses

**Figure 2:**
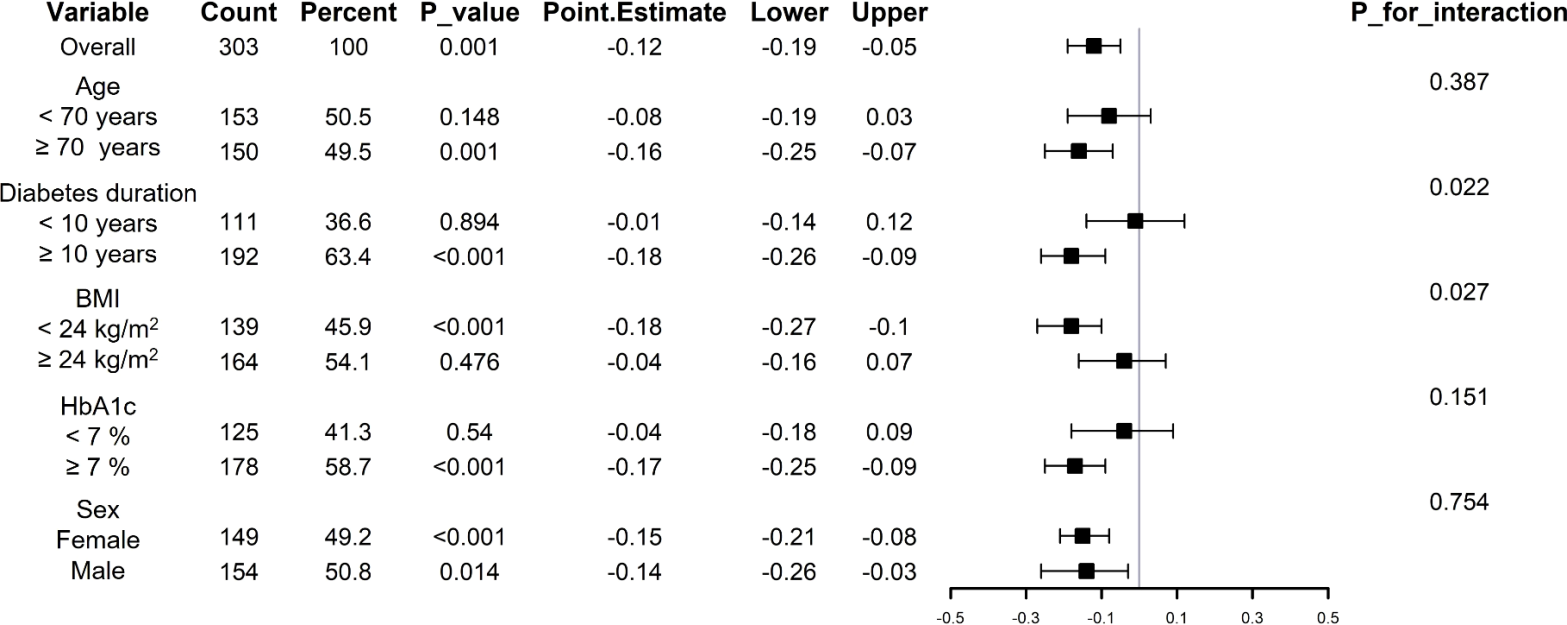
Forest plot of subgroup analyses for the association between CV and PMI. Squares represent standardized β coefficients for CV in each subgroup; horizontal lines indicate 95% confidence intervals. P values for interaction are shown on the right. All models were adjusted for age, sex, BMI, diabetes duration, HbA1c, albumin, hemoglobin, and serum creatinine (except for the stratification variable itself).

We then assessed whether the association between CV and PMI varied across different subgroups (**Figure 2**). After adjusting for the same set of covariates, the overall association remained significant (β = –0.12, 95% CI: –0.19 to –0.05, P = 0.001). Diabetes duration appeared to modify the relationship (P for interaction = 0.022). The inverse association was present in patients with diabetes duration ≥ 10 years (β = –0.18, P < 0.001) but not in those with shorter duration (β = –0.01, P = 0.894). BMI was also a significant effect modifier (P for interaction = 0.027). Among patients with BMI < 24 kg/m², CV showed a strong inverse association with PMI (β = –0.18, P < 0.001), whereas no such association was observed in those with BMI ≥ 24 kg/m² (β = –0.04, P = 0.476).

### Sensitivity analyses

We conducted several sensitivity analyses to test the robustness of our findings, all adjusting for the same covariates. As summarized in **Table 7**, the inverse association between GV parameters and PMI remained consistent across analyses—whether we excluded patients with extreme PMI values, excluded insulin users, or added nutritional markers (prealbumin, transferrin) to the model.

**Table 7:** Summary of sensitivity analyses for the association between glycemic variability parameters and PMI.

Sensitivity analysis	CV (β)	CV (95% CI)	CV (P)	SD (β)	SD (P)	MAGE (β)	MAGE (P)	TBR (β)	TBR (P)
Main analysis	-0.14	(-0.21, -0.08)	<0.001	-1.02	<0.001	-0.39	0.010	-0.18	<0.001
Excluding extreme PMI values	-0.13	(-0.20, -0.06)	<0.001	-0.96	<0.001	-0.35	0.019	-0.16	0.001
Excluding insulin users	-0.15	(-0.26, -0.04)	0.008	-1.10	0.013	-0.45	0.028	-0.21	0.004
Additional adjustment for prealbumin + transferrin	-0.13	(-0.20, -0.06)	<0.001	-0.94	<0.001	-0.32	0.036	-0.17	<0.001

All models adjusted for age, sex, BMI, diabetes duration, HbA1c, albumin, hemoglobin, and serum creatinine. Extreme PMI values defined as < 1st percentile or > 99th percentile.

### Reproducibility of PMI measurements

PMI measurements showed excellent reproducibility. The intraclass correlation coefficient was 0.94 (95% CI: 0.91–0.96) for intra-observer reliability and 0.91 (95% CI: 0.88–0.94) for inter-observer reliability, indicating good to excellent agreement (19).

## Discussion

In this retrospective analysis of 303 older adults with T2DM, we aimed to investigate whether CGM-derived glycemic variability was associated with CT-based pectoralis muscle mass. The main finding is that four GV parameters—CV, SD, MAGE, and TBR—were independently associated with lower PMI after adjusting for a range of confounders. Among these, CV explained the largest proportion of variance in PMI, so we used it as the primary indicator in subsequent analyses. The relationship followed a linear dose-response pattern and was more pronounced in patients with diabetes duration of 10 years or longer, as well as in those with BMI below 24 kg/m². Both factors showed significant effect modification. Sensitivity analyses suggested that these findings were fairly robust across different subgroups and model specifications.

The observed inverse association between glucose fluctuation and PMI is biologically plausible. Several mechanisms may explain why oscillating glucose levels damage skeletal muscle more than sustained hyperglycemia. One key pathway involves oxidative stress and mitochondrial dysfunction. *In vitro* and animal studies have shown that fluctuating glucose triggers greater reactive oxygen species production and mitochondrial damage than constant high glucose, leading to increased myocyte apoptosis and impaired muscle regeneration (21, 22). Another mechanism has to do with insulin signaling. Repeated glucose excursions may desensitize the PI3K/Akt/mTOR pathway, which is critical for muscle protein synthesis (23). A third mechanism involves inflammation: oscillating glucose levels can promote the release of pro-inflammatory cytokines like IL-6 and TNF-α, which activate ubiquitin-proteasome pathways and accelerate muscle protein breakdown (24). Notably, the significant association with TBR suggests that hypoglycemic episodes, which are not uncommon in insulin-treated patients, may contribute to muscle loss by triggering sympathetic nervous system activation or directly affecting myocyte metabolism (25).

Our findings are consistent with and extend the current understanding of the relationship between glycemic control and muscle mass. Several cross-sectional studies have reported an inverse relationship between HbA1c and skeletal muscle mass in people with diabetes (7, 26); however, HbA1c reflects mean glycemia over the preceding two to three months and does not capture day-to-day fluctuations. Recently, glucose variability has been recognized as an independent risk factor for diabetic complications, separate from mean glucose levels. With respect to sarcopenia specifically, higher glucose variability, assessed by self-monitored blood glucose, has been linked to lower appendicular skeletal muscle mass in older adults with diabetes (27). Our study adds a few things to this literature. First, we used CGM-derived parameters, which give a much more accurate picture of glucose fluctuations than self-monitoring. Second, we compared several GV metrics side by side—CV, SD, MAGE, and TBR—and found that CV accounted for the largest share of variance in PMI. Third, we identified diabetes duration and BMI as effect modifiers, which, to our knowledge, has not been reported previously.

Why did CV come out ahead of the other GV metrics? CV is calculated as (SD/MBG) × 100%, so it reflects glucose variability relative to mean glucose levels. It is less influenced by average glycemia, which makes it a preferred metric when comparing variability across individuals with different mean glucose levels (28). The higher adjusted R² we saw for CV suggests that this relative measure might capture the metabolic stress on skeletal muscle better than absolute measures like SD or MAGE. That is consistent with recent international consensus recommendations that CV is a core metric for glycemic variability in both clinical practice and research (11).

The finding that the association between CV and PMI was modified by diabetes duration and BMI has potential clinical implications. The association was only robust in patients with disease duration of 10 years or longer. This may suggest that the effects of glucose fluctuations on muscle accumulate over time, or it could reflect that patients with longer-standing diabetes tend to have more impaired β-cell function and greater insulin resistance, making them more susceptible to glucose fluctuation-induced muscle catabolism. Taken together, these findings indicate that stabilizing glycemic variability might be especially important in patients with long-standing diabetes, who are already at higher risk for sarcopenia.

The modifying effect of BMI is notable. The inverse association between CV and PMI was significant only in patients with BMI < 24 kg/m², consistent with the “obesity paradox” observed in aging and chronic disease populations (29, 30). One possible explanation is that adipose tissue may provide metabolic reserve or mechanical protection against muscle wasting. Alternatively, the null association in the higher BMI group may partly reflect a measurement limitation: CT-derived PMI does not distinguish intermuscular adipose tissue from muscle, which could attenuate the observed association. Regardless, this finding highlights that normal- or low-weight elderly T2DM patients may represent a particularly vulnerable subgroup, for whom optimizing glycemic stability could be especially important.

Several limitations should be acknowledged. The cross-sectional design precludes causal inference; reverse causality or residual confounding cannot be ruled out. The study was single-center and retrospective, which may introduce selection bias and limit generalizability. We assessed only muscle mass using CT-derived PMI, without muscle strength or physical performance measures—a limitation given that a definitive diagnosis of sarcopenia requires both (31). We were also unable to adjust for dietary intake, physical activity, or socioeconomic status, all of which can influence both glycemic control and muscle health. The study population consisted exclusively of Chinese patients, so findings may not extend to other ethnic groups. Although we performed extensive sensitivity analyses, unmeasured confounding cannot be entirely excluded. The opportunistic CT measurement of PMI used a single axial slice at T4 level, which may not fully capture whole-body muscle mass, though prior studies have validated its correlation with total skeletal muscle. Finally, the subgroup analyses were exploratory and should be interpreted cautiously given the multiple comparisons.

In this retrospective study of 303 elderly patients with T2DM, greater glycemic variability, particularly coefficient of variation, was independently associated with lower pectoralis muscle index. This association was more pronounced in patients with longer diabetes duration (≥ 10 years) and those with normal or low body weight (BMI < 24 kg/m²). Our findings suggest that in addition to achieving target HbA1c levels, stabilizing glycemic fluctuations may be an important consideration for reducing the risk of muscle loss in the aging diabetic population. Future prospective studies are warranted to confirm causality and evaluate whether interventions targeting glycemic variability can reduce the burden of diabetic sarcopenia.

## Data Availability

The raw data supporting the conclusions of this article will be made available by the authors, without undue reservation.

## Supplementary material

Supplementary Table 1 Extended laboratory and medication characteristics according to PMI tertiles. *Data are mean ± SD or n (%). P values from one-way ANOVA, Kruskal–Wallis, or chi-square test. DPP-4, dipeptidyl peptidase-4; SGLT-2, sodium–glucose cotransporter 2; GLP-1, glucagon-like peptide-1; AST, aspartate aminotransferase; ALT, alanine aminotransferase; BUN, blood urea nitrogen; eGFR, estimated glomerular filtration rate; NGAL, neutrophil gelatinase-associated lipocalin; TSH, thyroid-stimulating hormone.
