# Supplementary material for: Glycemic variability and muscle loss in elderly type 2 diabetes: insights from continuous glucose monitoring and chest CT in 303 patients
Source: Front Endocrinol (Lausanne). 2026 Jun 22;17:1863226. doi: 10.3389/fendo.2026.1863226 (PMC13333411; doi:10.3389/fendo.2026.1863226)
Supplement: Supplementary Table 1 — Extended laboratory and medication characteristics according to PMI tertiles. *Data are mean ± SD or n (%). P values from one-way ANOVA, Kruskal–Wallis, or chi-square test. DPP-4, dipeptidyl peptidase-4; SGLT-2, sodium–glucose cotransporter 2; GLP-1, glucagon-like peptide-1; AST, aspartate aminotransferase; ALT, alanine aminotransferase; BUN, blood urea nitrogen; eGFR, estimated glomerular filtration rate; NGAL, neutrophil gelatinase-associated lipocalin; TSH, thyroid-stimulating hormone. [file Table1.docx]

**Supplementary Table S1. Extended laboratory and medication characteristics according to PMI tertiles**

| **Variables** | **Total (n=303)** | **Lower tertile (n=101)** | **Middle tertile (n=101)** | **Upper tertile (n=101)** | ***P* value** |
| --- | --- | --- | --- | --- | --- |
| **Additional behaviors** |  |  |  |  |  |
| Alcohol consumption, n (%) | 6 (1.98) | 3 (2.97) | 1 (0.99) | 2 (1.98) | 0.600 |
| **Additional complications** |  |  |  |  |  |
| Diabetic peripheral neuropathy, n (%) | 238 (78.55) | 79 (78.22) | 79 (78.22) | 80 (79.21) | 0.981 |
| Diabetic peripheral vasculopathy, n (%) | 79 (26.07) | 26 (25.74) | 25 (24.75) | 28 (27.72) | 0.887 |
| **Additional medications** |  |  |  |  |  |
| Metformin, n (%) | 95 (31.35) | 34 (33.66) | 33 (32.67) | 28 (27.72) | 0.622 |
| Sulfonylureas, n (%) | 25 (8.25) | 10 (9.90) | 5 (4.95) | 10 (9.90) | 0.336 |
| DPP-4 inhibitors, n (%) | 47 (15.51) | 18 (17.82) | 16 (15.84) | 13 (12.87) | 0.620 |
| SGLT-2 inhibitors, n (%) | 173 (57.10) | 58 (57.43) | 52 (51.49) | 63 (62.38) | 0.293 |
| GLP-1 receptor agonists, n (%) | 50 (16.50) | 18 (17.82) | 15 (14.85) | 17 (16.83) | 0.846 |
| Thiazolidinediones, n (%) | 5 (1.65) | 2 (1.98) | 1 (0.99) | 2 (1.98) | 0.816 |
| α-Glucosidase inhibitors, n (%) | 102 (33.66) | 30 (29.70) | 36 (35.64) | 36 (35.64) | 0.587 |
| Glinides, n (%) | 26 (8.58) | 11 (10.89) | 8 (7.92) | 7 (6.93) | 0.579 |
| Statin, n (%) | 231 (76.24) | 75 (74.26) | 71 (70.30) | 85 (84.16) | 0.058 |
| **Additional laboratory** |  |  |  |  |  |
| Fasting C-peptide (ng/mL) | 2.45 ± 2.07 | 2.56 ± 2.57 | 2.43 ± 1.90 | 2.38 ± 1.64 | 0.970 |
| Postprandial 2h C-peptide (ng/mL) | 6.54 ± 6.18 | 6.40 ± 6.56 | 6.68 ± 5.81 | 6.55 ± 6.21 | 0.912 |
| Prealbumin (mg/L) | 212.63 ± 41.14 | 208.74 ± 43.31 | 216.65 ± 37.91 | 212.51 ± 42.04 | 0.443 |
| Transferrin (g/L) | 2.10 ± 0.34 | 2.10 ± 0.39 | 2.12 ± 0.35 | 2.09 ± 0.28 | 0.978 |
| Retinol-binding protein (mg/L) | 41.52 ± 10.70 | 40.96 ± 11.43 | 42.25 ± 9.99 | 41.35 ± 10.70 | 0.494 |
| Serum iron (μmol/L) | 13.97 ± 4.96 | 13.58 ± 4.64 | 13.80 ± 5.38 | 14.53 ± 4.84 | 0.251 |
| Total cholesterol (mmol/L) | 4.28 ± 1.20 | 4.27 ± 1.29 | 4.37 ± 1.17 | 4.22 ± 1.13 | 0.750 |
| Triglycerides (mmol/L) | 1.69 ± 1.01 | 1.64 ± 0.85 | 1.72 ± 1.05 | 1.72 ± 1.12 | 0.941 |
| LDL-C (mmol/L) | 2.52 ± 0.91 | 2.52 ± 0.97 | 2.56 ± 0.80 | 2.47 ± 0.96 | 0.620 |
| HDL-C (mmol/L) | 1.03 ± 0.25 | 1.03 ± 0.25 | 1.05 ± 0.25 | 1.01 ± 0.26 | 0.360 |
| AST (U/L) | 18.53 ± 9.58 | 18.65 ± 8.88 | 19.79 ± 12.48 | 17.16 ± 6.25 | 0.413 |
| ALT (U/L) | 19.03 ± 13.04 | 19.39 ± 13.03 | 19.64 ± 14.95 | 18.07 ± 10.91 | 0.916 |
| Total bilirubin (μmol/L) | 9.73 ± 4.21 | 9.50 ± 4.39 | 9.31 ± 4.09 | 10.38 ± 4.09 | 0.092 |
| BUN (mmol/L) | 6.28 ± 2.21 | 5.97 ± 1.92 | 6.52 ± 2.46 | 6.35 ± 2.20 | 0.321 |
| eGFR (mL/min/1.73 m²) | 102.11 ± 32.48 | 104.69 ± 33.68 | 99.83 ± 32.94 | 101.80 ± 30.88 | 0.590 |
| Cystatin C (mg/L) | 0.95 ± 0.30 | 0.97 ± 0.28 | 0.98 ± 0.37 | 0.92 ± 0.22 | 0.589 |
| β2-microglobulin (mg/L) | 2.86 ± 1.39 | 2.86 ± 1.38 | 3.00 ± 1.58 | 2.73 ± 1.19 | 0.469 |
| NGAL (ng/mL) | 136.69 ± 73.46 | 136.28 ± 66.18 | 132.96 ± 81.79 | 140.81 ± 72.08 | 0.273 |
| Serum uric acid (μmol/L) | 290.21 ± 98.20 | 282.79 ± 102.69 | 292.03 ± 96.43 | 295.80 ± 95.85 | 0.404 |
| TSH (mIU/L) | 2.69 ± 2.95 | 2.63 ± 3.58 | 2.65 ± 2.11 | 2.78 ± 3.01 | 0.255 |
| Calcium (mmol/L) | 2.22 ± 0.12 | 2.21 ± 0.12 | 2.23 ± 0.11 | 2.22 ± 0.14 | 0.412 |
| Phosphorus (mmol/L) | 1.20 ± 0.61 | 1.18 ± 0.21 | 1.27 ± 1.03 | 1.15 ± 0.19 | 0.609 |

*Data are mean ± SD or n (%). P values from one-way ANOVA, Kruskal–Wallis, or chi-square test. DPP-4, dipeptidyl peptidase-4; SGLT-2, sodium–glucose cotransporter 2; GLP-1, glucagon-like peptide-1; AST, aspartate aminotransferase; ALT, alanine aminotransferase; BUN, blood urea nitrogen; eGFR, estimated glomerular filtration rate; NGAL, neutrophil gelatinase-associated lipocalin; TSH, thyroid-stimulating hormone.
